# Ozone Synthesis Based on Dielectric Barrier Discharge Coupled Catalyst: Research Status and Future Perspectives

**DOI:** 10.3390/nano16040238

**Published:** 2026-02-12

**Authors:** Meng Li, Li Xu, Lei Wang, Wei Zhang, Yang Yang, Zhen Wang, Dapeng Wu, Kai Jiang

**Affiliations:** 1 International Joint Laboratory on Key Techniques in Water Treatment, Henan Province, School of Environment, Henan Normal University, Xinxiang 453007, China; 2020010@htu.edu.cn (M.L.);; 2 Faculty of Preschool Education and Arts, Henan Logistics Vocational College, Xinxiang 453514, China; 3 Novel Energy Materials & Catalysis Research Center, Shanwei Innovation Industrial Design & Research Institute, Shanwei 516600, China; 4 College of Environmental Sciences and Engineering, Dalian Maritime University, Dalian 116026, China

**Keywords:** ozone synthesis, dielectric barrier discharge, catalyst, synergistic catalysis

## Abstract

Efficient ozone synthesis has always been the pursuit of ozone workers and the foundation for the industrial application of ozone reactors. Recently, with continuous breakthroughs in materials and catalyst research, as well as the rapid development of advanced characterization technologies, introducing catalysts into dielectric barrier discharge (DBD) to build a DBD–catalyst coupled system has developed into an advanced means of improving ozone synthesis and attracted widespread attention. This review aims to provide a systematic summary for the research status of the DBD–catalyst coupled system in the field of ozone synthesis. Firstly, the structure of DBD reactors (type and shape of the electrode, etc.), catalyst types and the coupling method of DBD and catalysts (such as catalyst packing, catalyst coating/film) for the DBD–catalyst coupled system are discussed. Subsequently, the relevant mechanisms involving plasma gas-phase reactions and gas–solid interface reactions for elevating discharge ozone synthesis through coupling catalysts with DBD are summarized and analyzed. Afterwards, the research status of the DBD–catalyst coupled system in the field of ozone synthesis is surveyed. At present, the optimal ozone synthesis performance of the reactor with packed catalyst in air plasma (γ-Al_2_O_3_ sphere) is 0.96 g/Nm^3^ and 103 g/kWh, and in oxygen plasma (SiO_2_ particle) is 130 g/Nm^3^ and 91 g/kWh, respectively. For the reactor coupled with a catalyst coating, the performance reaches 19.3 g/Nm^3^ and 320 g/kWh in oxygen plasma (TiO_2_). Then, advanced plasma parameter detection techniques (i.e., optical emission spectroscopy and two-photon absorption laser-induced fluorescence) are expatiated to observe the changes in plasma parameters within the discharge system and then provide strong support for further in-depth research and analysis of the enhancement mechanism of coupling catalysts on ozone synthesis. Finally, a short conclusion, together with the current challenges and future opportunities of the DBD–catalyst coupled system in improving ozone synthesis, are proposed.

## 1. Introduction

Ozone (O_3_) has extremely strong oxidation capacity (the redox potential is 2.07 V), and can achieve effective oxidation removal of organic pollutants (neonicotinoid insipides [1,2], toluene [3], Bisphenol-A [4], ibuprofen [5], etc.) and rapid inactivation of bacteria, fungi, spores, viruses and other microbial pathogens in the environment (SARS-CoV-2 virus [6,7], *E. coli* [8,9], *S. aureus* [10], *F. fujikuroi* [11], etc.) without special conditions. Generally, in addition to the direct oxidation of pollutants by O_3_ molecules, some radicals (such as hydroxyl radical, superoxide radical, and peroxide ion) that are produced by O_3_ decomposition under different ambient humidities can also exhibit a strong oxidative decomposition ability against organic pollutants and microbial pathogens from the environment [12,13,14]. It is gratifying that the absence of toxic by-products indicates that the use of O_3_ does not cause secondary pollution to the environment. In view of this, O_3_ has been widely used as a green strong oxidant to solve pollution or production problems in industrial, agricultural and medical fields, such as drinking water and sewage treatment, exhaust-gas purification, aquaculture, food preservation and utensil/machinery cleaning or disinfection [1,15,16,17,18]. Therefore, in terms of the application of O_3_, its efficient synthesis not only can achieve energy conservation, but also has important significance for improving industrial and agricultural production and the quality of human life. However, how to acquire efficient O_3_ synthesis, that is, obtaining a high O_3_ concentration with low energy-consumption, is still a key challenge faced by O_3_ researchers at present.

Given the difficulties in utilizing O_3_ generated under natural conditions (such as photochemical reactions from sunlight and electrical sparks from lightning), O_3_ in practical applications is generally synthesized artificially. At present, the main methods for artificially synthesizing O_3_ include ultraviolet radiation [19], electrolysis [20,21], gas discharge, etc. Among these methods, gas discharge is the most used (such as glow discharge [22,23], nanosecond pulsed streamer discharge [24,25], corona discharge [26,27], dielectric barrier discharge (DBD) [28,29,30,31]). However, the implementation of glow discharge and nanosecond pulsed streamer discharge commonly requires a vacuum/low-voltage environment and nanosecond pulsed power supply, respectively, which invisibly increases operating costs and limits their application. Although corona discharge can be achieved at atmospheric pressure, it has lower O_3_ synthesis efficiency. In contrast, DBD not only has a wide range of working conditions, simple operation and lower costs, but also can generate a large-area nonthermal plasma, providing significant benefits for O_3_ synthesis reactions. Therefore, DBD typically outperforms other gas discharge methods in O_3_ synthesis efficiency and commercialization potential, garnering great attention from O_3_ researchers.

Although DBD possesses good performances in O_3_ synthesis, its experimental synthesis efficiency still falls drastically short of the theoretical value (about 1226 g/kWh) [32,33,34], and the O_3_ concentration achieved at higher synthesis efficiencies often remains inadequate. For instance, Šimek et al. [35] obtained an O_3_ concentration of only 10 g/Nm^3^ at the highest O_3_ synthesis efficiency of 170 g/kWh; Malik et al. [36] and Yuan et al. [37] achieved O_3_ synthesis efficiency of 250 g/kWh and 174.4 g/kWh, respectively, with corresponding O_3_ concentrations as low as 10 g/Nm^3^ and 22.6 g/Nm^3^, respectively. For this reason, scholars have implemented substantial improvements to DBD for promoting discharge O_3_ synthesis, then achieving a high O_3_ concentration and efficiency. So far, multiple methods have been proven to effectively promote the O_3_ synthesis of DBD: (i) Adopting superior geometric parameters for DBD reactors to enhance the discharge, such as thinner dielectric layers [38,39], narrower discharge gaps and a dielectric layer with higher dielectric constants [40,41,42,43,44]; (ii) Employing an efficient DBD mode, such as double surface discharge [38,39], hybrid discharge [45,46,47,48,49], or sliding discharge [36,50]; (iii) Employing advanced gas-flow modes to prolong the gas–plasma interaction duration (ensuring more complete interaction), such as the gas-flow mode shifts from linear to curved flow [51], or from simultaneously passing through multiple discharge gaps to sequentially passing through each gap (only for DBD reactors with multiple discharge gaps) [38]; (iv) Using nanosecond pulse power supply for the reactor or increasing the power supply frequency to reduce thermal and dielectric losses [24,52]; (v) Equipping the reactor with a cooling device to suppress the thermal decomposition of O_3_ [35,53,54]; (vi) Introducing an external magnetic field into the discharge gap of DBD to improve the electron temperature [55,56]. In addition to the aforementioned methods, with continuous breakthroughs in materials and catalyst research, as well as the rapid development of advanced characterization technologies in recent years, combining appropriate catalysts with DBD to establish a DBD–catalyst coupled system has also evolved into an alternative advanced technology for enhancing O_3_ synthesis.

For the DBD–catalyst coupled system, the catalyst is the critical factor driving the improvement of O_3_ production, usually located in the plasma of DBD in the form of packing or coating/film. This is because the presence of catalysts can not only alter the plasma process in DBD to accelerate O_3_ synthesis reactions, but also positively influence O_3_ synthesis through changing the surface properties and structure of catalysts during its interaction with high-energy electrons and other active particles in the plasma. The specific manifestations are as follows: (i) Enhancement of electric field strength. Catalysts can generate locally enhanced non-uniform electric fields within DBD, which can increase the concentration of high-energy electrons and active particles in the plasma [57,58]; (ii) Changes in plasma discharge modes. The catalyst can increase the discharge current density, making DBD more intense and stable. When catalysts are packed, the discharge mode of DBD transforms from the traditional filamentary discharge to partial discharge and surface discharge within the reduced discharge volume fraction in the gap [59,60,61]; (iii) The adsorption characteristics of the catalyst prolong the plasma reaction time. The adsorption of oxygen molecules and other active species on the catalyst surface prolongs their residence time in DBD, making reactions for O_3_ synthesis more complete [60,62]; (iv) DBD can significantly increase the surface area of catalysts by reducing its particle size, while also creating more defects (such as oxygen vacancies) on its surface, thereby providing more surface catalytic active centers and expediting gas–solid interface reactions [58,63]; (v) The photocatalytic effect generated by the catalyst during the plasma process can also affect the discharge O_3_ synthesis [64,65,66].

Consequently, when constructing a DBD–catalyst coupled system, through regulating the physical and chemical properties of catalysts (such as material, shape, surface morphology and chemical state, surface defect degree, photo responsivity, etc.) based on advanced catalyst preparation technology, it is easy to acquire the positive effects of catalysts on the discharge characteristics, plasma parameters, plasma gas-phase reactions and gas–solid interface reactions of DBD, thereby significantly improving O_3_ synthesis [67]. The DBD–catalyst coupled system demonstrates significant potential for enhancing O_3_ synthesis and has emerged as a prominent research focus. However, up to now, there has been no comprehensive review of a DBD–catalyst coupled system used in O_3_ synthesis. Chen et al. [68] reviewed the progress of DBD with packing dielectric pellets for O_3_ synthesis, but nearly two decades have elapsed since then, and critically, their review did not address the nanocatalyst coating/film. Considering the increasing attention of O_3_ technology in controlling environmental pollution and the breakthrough progress of catalysts in enhancing discharge O_3_ synthesis in recent years, it is very necessary to conduct a systematic and in-depth review of DBD–catalyst coupled system applied in O_3_ synthesis, providing reference for further research and exploration by O_3_ researchers in the future.

Herein, we review the research status of a DBD–catalyst coupled system in the field of O_3_ synthesis. Firstly, the DBD–catalyst coupled system is introduced, including the structure of DBD reactors, catalyst types and the coupling method of DBD and catalysts. Meanwhile, the relevant mechanisms of boosting DBD O_3_ synthesis via coupling catalysts are summarized and discussed. Then, the research status of the DBD–catalyst coupled system used in O_3_ synthesis is focused. Afterwards, advanced plasma parameter detection techniques (such as two-photon absorption laser-induced fluorescence) are elaborated, aiming to offer strong support for further in-depth research and analysis of the enhancement mechanism via coupling catalysts on O_3_ synthesis. Finally, the current challenges and prospects of the DBD–catalyst coupled system in enhancing O_3_ synthesis are summarized.

## 2. DBD–Catalyst Coupled System

### 2.1. DBD Reactor

**Figure 1:**
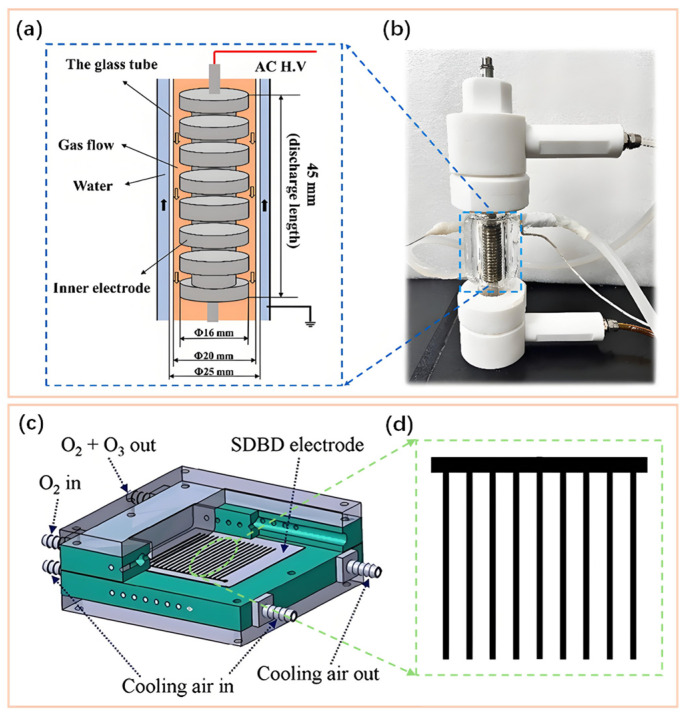
Typical cylindrical DBD reactor: Schematic diagram (**a**) and actual picture (**b**) (Reproduced with permission from reference [70]); typical planar DBD reactor (**c**) (Reproduced with permission from reference [35]); fence-like electrode (**d**) (Reproduced with permission from reference [65]).

DBD was first systematically documented by German scientist Ernst Werner von Siemens in 1857 [69]. To date, after over a century of development spanning four stages: initial discovery and observation, theoretical germination and industrialization, theoretical deepening and the concept emergence of “low-temperature plasma”, and explosive application growth, DBD has now gained widespread adoption in laboratories, industry and agriculture settings as a convenient, mature and reliable O_3_ synthesis technology [70,71,72]. However, to improve the energy efficiency and industrial application of DBD O_3_ reactors, extensive research and continuous improvement on the structure of DBD reactors are ongoing. Typically, DBD reactors can be broadly classified into tubular and planar types based on their geometric shapes, as illustrated in Figure 1. In addition to conventional metal tubes/plates as electrodes, researchers have also designed electrodes in various forms (mesh, spiral and fence-like, etc.) [28,71,73,74,75] and materials (such as water, aqueous solution) [70,76,77]. Jodzis et al. [78], Malik et al. [36,50,79], and Chebbah et al. [51] employed mesh, fence-like and serpentine-shape metal electrodes, respectively, in DBD O_3_ synthesis. While Yao et al. [76] and Yuan et al. [70] replaced the conventional metal grounding electrodes with NaCl aqueous solutions and water, respectively, which simultaneously enable electrical conductivity and cooling functions. Based on these diverse electrode forms and their combinations, a range of DBD modes have been developed, including volume discharge, surface discharge, coplanar discharge, sliding discharge and hybrid discharge [45,47,48,49,80,81,82,83,84]. In addition, the dielectric layer has also developed various materials (quartz, glass, ceramic, mica, etc.). For example, the dielectric layer of Malik et al. and Wu et al. employed quartz [77], whereas Jin et al. adopted glass [75]. Li et al. conducted comparative experiments of O_3_ synthesis using Al_2_O_3_, ZrO_2_ and AlN dielectric layers [38,45,58,67]. The continuous innovations in reactor design and material selection have laid a robust foundation for advancing the development of DBD technology and O_3_ synthesis fields.

#### 2.1.1. Cylindrical DBD Reactor

**Figure 2:**
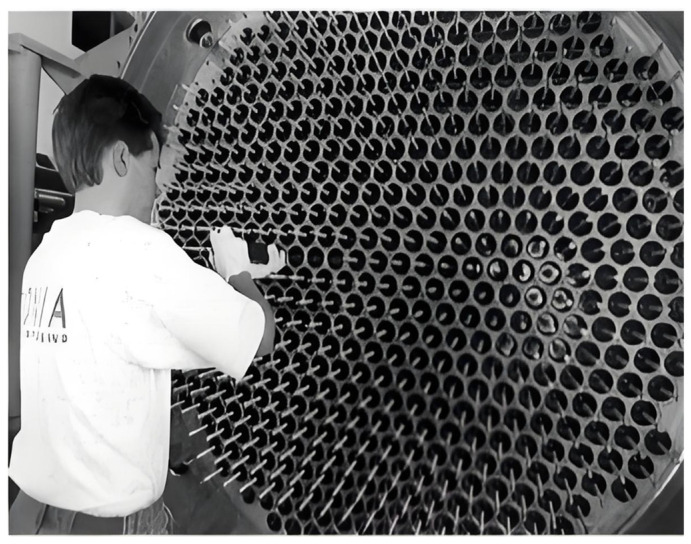
Integrated large-scale O_3_ generator (Reproduced with permission from reference [85]).

The cylindrical DBD reactor representing the earliest geometry for commercial O_3_ generators (as shown in Figure 1a,b) is commonly used for large-scale industrial O_3_ synthesis (O_3_ production of over 1 kg/h). This is primarily attributed to its following characteristics [69,85,86]: (i) Mature O_3_ synthesis technology [85,87]; (ii) High mechanical strength. Tubular structures, particularly glass tubes, possess superior resistance to internal and external pressure differentials and offer enhanced sealing and manufacturability [85]; (iii) Uniform electric field. The uniform distribution of the electric field within the tubular reactor facilitates its calculation and control, thereby enabling the stable discharge and extended dielectric lifetime [88]; (iv) Easy to equip with cooling measures. Circulating cooling water (or other cooling liquids) flows through the metal electrode tubes (the grounded tubes or central electrode tubes), suppressing O_3_ thermal decomposition and improving O_3_ production via facilitating heat dissipation [77,89]; (v) Easy to integrate and extend. Connecting multiple discharge tubes in parallel to form an “O_3_ generator tube bundle” makes it relatively easy to scale up production capacity [85]. For the integrated large-scale O_3_ generators, the length of a single discharge tube can exceed 3 m, and its O_3_ production usually exceeds 1 kg/h. Figure 2 displays the diagram of the integrated large-scale O_3_ generator.

Currently, the commercial large-scale O_3_ generator developed by Wedeco Company (Bexbach, Germany) has an O_3_ production of 100 kg/h and O_3_ synthesis efficiency of 125 g/kWh. While the O_3_ production of the large-scale O_3_ generator produced by Ozonia (Zürich, Switzerland) can reach up to 250 kg/h with O_3_ synthesis efficiency of 100 g/kWh. In China, the O_3_ production of the produced large-scale O_3_ generator by Nicoler Company (Shanghai, China) is 170 kg/h; the O_3_ generator from Qingdao Pioneerep Company (Qingdao, China) with dimensions of 7.2 m (length) × 4.8 m (width) × 3 m (height) possesses the O_3_ production of 100 kg/h in O_2_ discharge and 50 kg/h in air discharge; also, when adopting the dimensions of 7.8 m (length) × 5 m (width) × 3.5 m (height), the O_3_ generator from Qingdao Guolin Company (Qingdao, China) has a higher O_3_ production (200 kg/h in O_2_ discharge and 60 kg/h in air discharge) compared to Qingdao Pioneerep Company (Qingdao, China). These large-scale O_3_ generators with high O_3_ production effectively meet the substantial O_3_ demand in industrial applications to a certain extent; for example, printing and dyeing wastewater treatment, municipal sewage treatment and exhaust gas purification of coal-fired power plants [6,14,15,16,18]. In general, a large-scale O_3_ generator weighs several tons and requires custom-built equipment rooms, resulting in large land footprints and high investment costs. Although the large-scale O_3_ generator boasts a high O_3_ production, it still suffers from high energy consumption and low O_3_ synthesis efficiency (<200 g/kWh) [36,90]. Therefore, further improvements in O_3_ synthesis technology are required.

#### 2.1.2. Planar DBD Reactor

The planar DBD reactor has gradually matured with the advancement of new dielectric materials such as high-performance ceramics and specialty glass, as well as precision machining technologies for reactors [35,37,51,81,91]. Consequently, the commercial application of planar reactors in O_3_ synthesis occurred slightly later than that of tubular reactors. Figure 1c,d display their typical structures. In recent years, extensive research has been conducted on O_3_ synthesis of the planar DBD reactor with different structures, electrode shapes and electrode compositions [80,81,92,93]. Compared to tubular DBD, planar DBD is more commonly applied for developing efficient small- and medium-scale O_3_ generators [38,39]. On the one hand, large-area and uniform cooling is not easy to implement in planar DBD reactor. On the other hand, the ultra-thin (thickness as low as 0.25 mm) dielectric plate (such as ceramic) in planar DBD with superior dielectric performance (high dielectric constant, low dielectric loss and thermal expansion coefficient, etc.) is more suitable for small-scale processing [38,45]. With the advancement of society and the enhancement of people’s awareness of safety, adopting O_3_ technology to resolve common pollution issues in daily life (e.g., purifying the air inside small rooms and cars, purifying water quality in small-scale water plants, disinfection of bowls and chopsticks.) also has become increasingly important, so small- and medium-scale O_3_ generators are needed [39,67]. Recently, the small-scale O_3_ generator (0.6 m (length) × 0.6 m (width) × 0.3 m (height)) manufactured by Anseros Company (Stuttgart, Germany) obtains the highest O_3_ concentration of 300 g/Nm^3^, efficiency of 100 g/kWh and production of 120 g/h in O_2_ discharge, respectively. The mobile small-scale O_3_ generator (0.4 m (length) × 0.2 m (width) × 0.5 m (height)) produced by Ruiqing Company (Shenzhen, China) can achieve the highest O_3_ concentration and production of 25 g/Nm^3^ and 30 g/h, respectively, under air discharge. As indicated by the above analysis, for on-site applications with space constraints and requiring flexibility, the medium- and small-scale O_3_ generator possessing the features of being portable and easy-to-use, low-investment and high-efficiency is more practical.

In the context of current global energy shortages and the volatility of renewable energy sources, it is still essential to conserve and use energy reasonably. Therefore, regardless of the structure adopted by the DBD reactor and the application field of O_3_, maximizing O_3_ synthesis under limited electricity supply can not only save operating costs for enterprises, but also be the goal pursued by O_3_ researchers. The selection of an advanced DBD reactor can lay a solid foundation for the construction of the DBD–catalyst coupled system for strengthening O_3_ synthesis.

### 2.2. Catalyst and Its Coupling Method with DBD

#### 2.2.1. Catalyst

For the DBD–catalyst coupled system, the selection of the catalyst is of great importance, as it largely determines the catalytic performance of the coupled system. Up to now, oxide catalysts, such as TiO_2_, Al_2_O_3_, ZrO_2_ and SiO_2_, have been mostly used internationally to elevate DBD O_3_ synthesis [53,62,65,94,95,96]. Though noble-metal catalysts (Pt, Pd, Au, Ag, etc.) are recognized to have better catalytic activity than oxide catalysts, they are frequently employed in research on pollutants degradation such as volatile organic compounds (toluene, benzene, formaldehyde, etc.) [97,98,99,100,101]. Kim et al. [102] loaded Ag, Ni, Pt and Pd onto three different supports of *γ*-Al_2_O_3_, TiO_2_ and zeolite, and investigated the degradation performance of these catalysts for toluene and benzene in a DBD reactor. The study found that when the Ag-loading amount was lower, the degradation efficiency for both benzene and toluene was superior. Huu et al. [103] studied the oxidation of low concentrations of methane, propylene and toluene in air using plasma coupled with Pd/γ-Al_2_O_3_ catalyst under atmospheric pressure. The results indicate that the plasma coupled catalyst system can significantly improve the conversion rate of volatile organic compounds. Meanwhile, due to the synergistic effect between the plasma and catalyst, the formation of by-products such as formaldehyde, formic acid and O_3_ is significantly reduced. In addition, Zhu et al. [97,98,104] have also demonstrated in many studies that the coupled system of noble-metal Au or Ag supported nanocatalysts with DBD not only has high removal-efficiency for toluene, but also effectively inhibits the generation of O_3_.

Based on the application research of the DBD–catalyst coupled system, it can be concluded that simply increasing the catalytic activity of catalysts does not necessarily favor O_3_ synthesis, as it is also closely related to the catalytic selectivity of the catalyst during the catalytic process and the inherent properties of O_3_. Due to the unstable and easily decomposable nature of O_3_ [14,105], the strong catalytic performance of noble-metal catalysts often hinders O_3_ synthesis. This may not only be related to the highly active species generated by the interaction of plasma with noble-metal catalysts that are favorable for O_3_ decomposition [67] but may also be assigned to the dominance of O_3_ decomposition reaction in the gas–solid interface reaction over the surface of noble-metal catalysts [67,104]. In contrast, oxide catalysts are not only inexpensive, but also have relatively mild catalytic activity, which can facilitate O_3_ synthesis reactions during the interactions of plasma with oxide catalysts. In the experiment of enhancing DBD O_3_ synthesis via coupling catalysts, Pekárek et al. [65,66] adopted the oxide catalyst of TiO_2_, while SiO_2_ and Al_2_O_3_ were, respectively, used by Jodzis et al. [106] and Al-Abduly et al. [94]. They all achieved an improvement in O_3_ synthesis.

#### 2.2.2. Coupling Method

**Figure 3:**
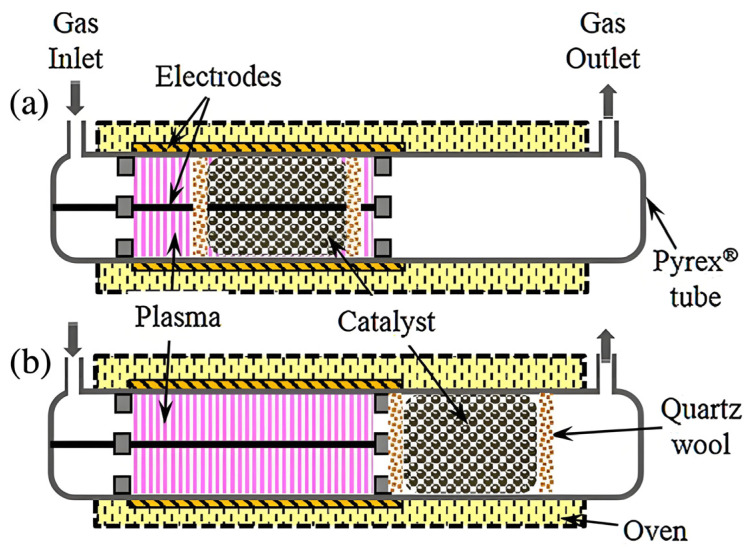
Schematic diagram of IPC (**a**) and PPC (**b**) (Reproduced with permission from reference [103]).

Catalytic plasma systems can be classified into two types based on the placement of the catalyst within the reactor (see Figure 3): in-plasma catalyst (IPC) system and post-plasma catalyst (PPC) system [103,107]. In the IPC system, the catalyst is located within the plasma zone, enabling direct contact and interaction with the plasma, then resulting in a strong synergistic effect [64,108,109]. So, the catalytic performance of the IPC system surpasses that of the plasma or catalyst used individually. In contrast, the catalyst in the PPC system is positioned outside the plasma zone at the rear end of the system, meaning that the catalyst does not come into direct contact with the plasma [110]. This indicates that, in the PPC system, the catalyst and plasma exhibit relatively weak interactions, as the lifetimes of high-energy electrons and reactive radicals in plasma are typically short, making it difficult to reach the surface of the catalyst positioned at the rear end of the PPC system. At present, the PPC system is commonly used for the degradation of organic compounds, but its application in O_3_ synthesis is rare. This is because the PPC system can better control the generation of plasma by-products, and the replacement and utilization of its catalysts is very convenient. For example, in the degradation of volatile organic compounds, the plasma first pretreats and partially mineralizes the volatile organic compounds. Then, the undegraded volatile organic compounds, converted by-products and generated O_3_, along with the gas flow, come into contact with the catalyst at the rear end of the PPC system and is completely mineralized and degraded via a catalytic ozonation process [110]. In contrast, the IPC system has been widely adopted for O_3_ synthesis due to its efficient O_3_ synthesis reactions.

**Figure 4:**
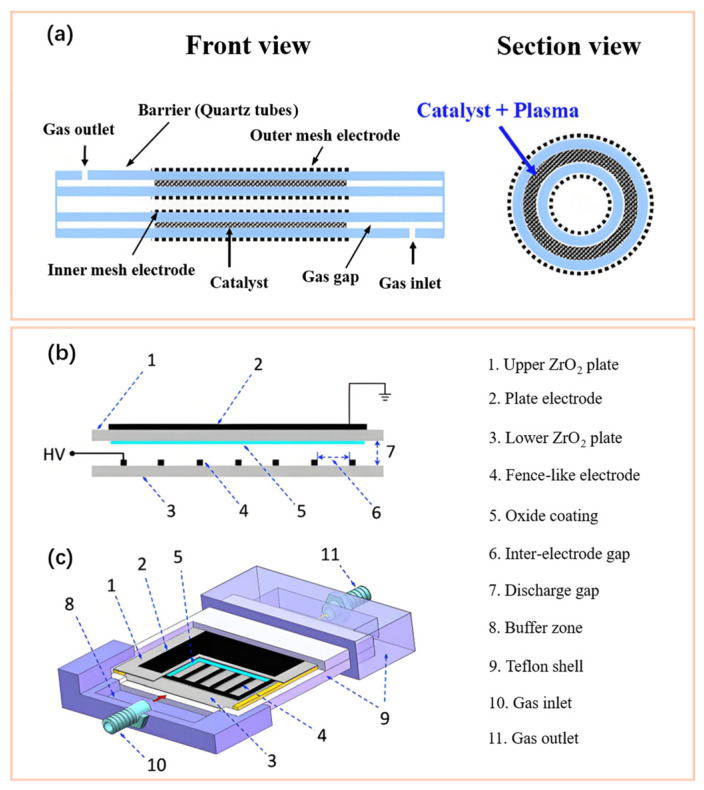
The cylindrical DBD packed with oxide beads (**a**) (Reproduced with permission from reference [60]); the planar DBD coupled with oxide coating: cross-sectional view of electrode configuration (**b**) and schematic diagram of the reactor (**c**) (Reproduced with permission from reference [67]).

In O_3_ synthesis, there are two primary methods for coupling oxide catalysts with the DBD reactor to build an IPC system. One method is to pack oxide catalysts into the discharge gap of the DBD reactor and then construct a packed DBD reactor [53,62,94,106], as shown in Figure 4a. In this method, the packed catalyst typically possesses different shapes. Across all studies, granular catalysts with a relatively large particle size are the most used. The oxide catalyst packed into the DBD reactor by Chen et al. used Al_2_O_3_ pellets with a particle size of 2, 5, and 10 mm, and glass beads with a particle size of 2, 3, and 5 mm, respectively [111]. The irregular glass and SiO_2_ with a particle size of about 1 mm were used as packing catalysts by Ni et al. [53] and Schmidt-Szałowski et al. [95], respectively. The Al_2_O_3_ pellets with a particle size of 2–3 mm were applied as packing catalysts by Al-Abduly et al. [94]. In addition, quartz fiber can also be used as a packing catalyst. For instance, when constructing the packed DBD reactor, Zeng et al. adopted the packing catalyst of pure quartz fibers and the quartz fibers loaded with SiO_2_ nanoparticles, respectively [62]. The advantage of this coupled system is that, during the discharge process, copious microdischarges can be produced on the surface of catalysts, stimulating the formation of higher local electric-field strength and more reactive species around catalysts, thereby accelerating plasma reactions and O_3_ synthesis [53,109,112]. Another method is to introduce a nanocatalyst coating/film into the DBD reactor by placing them over the inner surface of the reactor wall, dielectric layer or electrode [65,113,114], ensuring sufficient contact and interaction between the nanocatalyst coating/film and plasma during the discharge process (see Figure 4b,c). For example, Li et al. [58] placed a ZnO coating over the inner surface of a dielectric plate in a planar reactor. Wu et al. [115] coated a TiO_2_ film over the surface of a dielectric tube in a cylindrical reactor. The presence of the nanocatalyst coating/film can not only enhance the discharge to facilitate plasma reactions, but more importantly, during its sufficient interaction with plasma, the strong gas–solid interface reactions over the surface of the nanocatalyst coating/film can be induced. Moreover, regardless of the coupling method used, when the emission spectra from plasma matches the bandgap width of catalysts, the photocatalytic effect can be induced on the catalyst surface, thereby favoring the enhancement of catalytic performance for the DBD–catalyst coupled system as well [66,116,117].

In summary, the physical structure of the oxide catalyst (particle size, shape, etc.) and the discharge gap of the reactor chiefly determine the coupling method of a DBD and the oxide catalyst. Generally, a large discharge gap for the reactor is needed when catalysts and a DBD are coupled through a packing method, and the catalysts typically have relatively large dimensions (particles, fibers, etc.). In comparison, a DBD can achieve coupling with a nanocatalyst coating/film under extremely small discharge gaps in the reactor, as the thickness of the catalyst coating/film is commonly below the millimeter level.

## 3. Mechanism of Catalyst Enhanced DBD O_3_ Synthesis

As we know, the process of discharge O_3_ synthesis is very complex, involving a large number of plasma physicochemical reactions such as discharge ionization and ion recombination (see Table 1). To the discharge reactor without catalyst, the plasma gas-phase reaction in the discharge gap of the reactor dominates O_3_ synthesis. In pure O_2_ discharge, the O_3_ synthesis mainly originates from reactions R1-R5. Accordingly, the mechanism of O_3_ synthesis can be simply described as [35,57,118,119,120,121]: After applying high voltage, a strong electric field generates the plasma containing plentiful active species (such as high-energy electrons, ions, free radicals and excited molecules) in the discharge gap of reactor; O_2_ are decomposed into O within the plasma through collision reactions with high-energy electrons (R1), and then synthesized into O_3_ through a three-body collision process (R2); Meanwhile, these synthesized O_3_ would also undergo decomposition through collisions with some active species such as electrons and O (R3–R5). The above reaction process achieves equilibrium between the synthesis and decomposition of O_3_ in gas discharge. While in the air discharge, besides reactions R1–R5, many nitrogen-active species (N, N^+^, N_2_^+^, etc.) also participate in the complex reactions of O_3_ synthesis [81,122]. Therefore, during this O_3_ synthesis process, a series of by-products of nitrogen oxides are also formed, such as NO, N_2_O, NO_2_ and N_2_O_5_. However, these nitrogen oxides can decompose O_3_ through reaction Equations R6–R8, which can also provide additional O for the three-body collision reaction of O_3_ synthesis via triggering reaction R9–R11, then acquiring the equilibrium of O_3_ synthesis and decomposition with the participation of nitrogen species.

**Figure 5:**
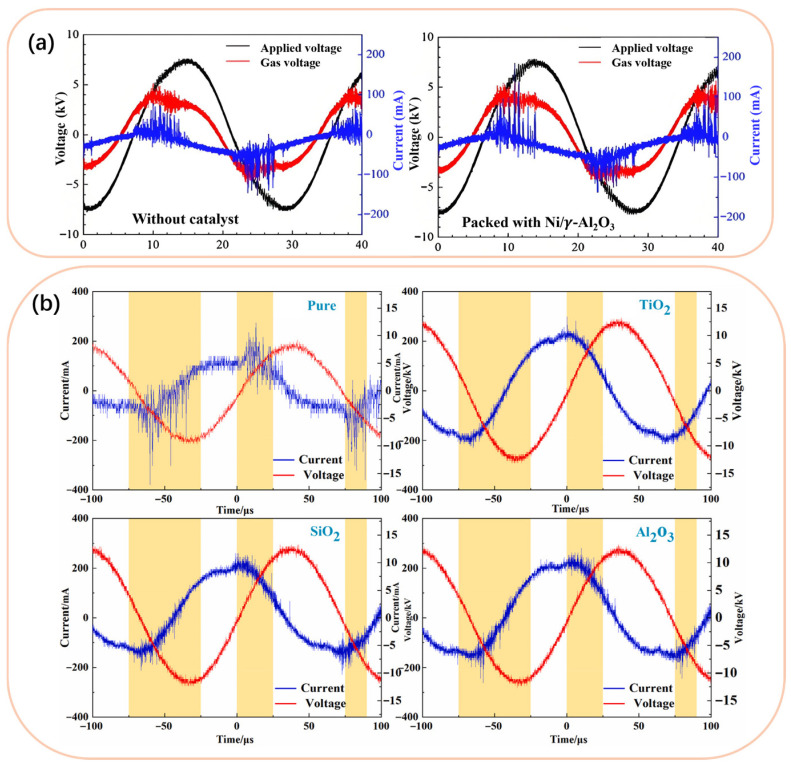
Electrical signals for DBD before and after packing catalyst (**a**) (Reproduced with permission from reference [60]) and coating catalyst (**b**) (Reproduced with permission from reference [61]).

**Figure 6:**
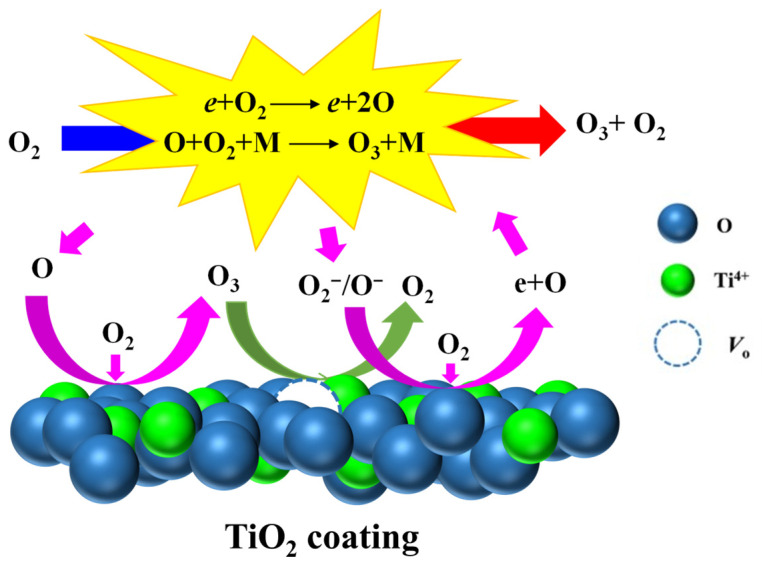
Schematic diagram of O_3_ synthesis reactions in DBD coupled with TiO_2_ coating (Reproduced with permission from reference [67]).

When the catalyst is introduced into DBD, as the discharge mode of DBD changes (see Figure 5), the O_3_ synthesis in the DBD–catalyst coupled system is mainly formed by two types of reactions, and the reaction mechanism is displayed in Figure 6. One is the gas-phase reaction in the discharge gap mentioned above, and the other is the strong gas–solid interface reactions that occurred over the catalyst surface [58,109]. Compared to DBD alone, the presence of catalysts can make DBD more intense and uniform, allowing it to receive more energy injection under the same conditions, then forming a strong local electric field strength in the discharge gap and inducing the production of more high-energy electrons and active particles, therefor significantly promoting plasma gas-phase reactions and O_3_ synthesis in the discharge gap [67,94]. For instance, Li et al. [67] improved the microdischarge current density of DBD by adding a TiO_2_ coating onto the surface of the dielectric layer, and simultaneously transformed the asymmetric microdischarge current pulses into symmetric ones. Zeng et al. [62] found that introducing quartz fiber into the plasma reactor significantly improves the discharge intensity and uniformity of the plasma. These results imply that the presence of a catalyst makes the discharge more uniform, thus facilitating plasma physicochemical reactions for O_3_ synthesis [67].

Furthermore, the strong gas–solid interface reaction on the catalyst surface also plays a vital role in O_3_ synthesis. This should be credited to the following reasons: (i) The rough surface of the coating gives it a much larger specific surface area and plentiful active or catalytic sites relative to the dielectric plate [58,96]; (ii) In situ plasma promotes the interaction between reactive oxygen species and active or catalytic sites [61,96]; (iii) A high electron-transfer rate at the gas-coating interface [67]; (iv) The photocatalytic effect generated by the photocatalyst during the plasma process [64,116]. Concretely, taking the DBD reactor coupled with the catalyst coating/film as an example, during the discharge process, the massive active or catalytic sites over the catalyst coating/film adsorb O_2_ and O easily from the gas-phase of the discharge gap around the coating/film surface, then expediting O_3_ formation through gas–solid interface reactions R12 and R13 [12]. Simultaneously, the collision of O_2_^−^ and O^−^ with the catalyst coating/film via reactions R14 and R15 can accelerate the reactions (R1) and (R12 and R13), conversely [45]. In gas-phase reactions, “M” in reaction (R2) representing the third-body collision partner mainly refers to active oxygen particles in O_2_ plasma. However, when using the catalyst coating/film, the coating/film in the DBD–catalyst system can also act as “M” in O_3_ synthesis via R16 due to its large specific surface area [30,44]. According to the studies from Chen et al. and Ni et al. [10,16], the catalyst coating/film can favor the conversion of O(^1^*D*, 1.94 eV) to O(^3^*P*, 0 eV), which weakens the decomposition of O_3_ by O(^1^*D*, 1.94 eV). Additionally, if the bandwidth of the catalyst coating/film coupled within DBD matches the spectra emitted by the plasma, the photocatalytic effect can be induced over the catalyst surface and have a positive impact on O_3_ synthesis as well [64,65,66,116]. Lu et al. and Capp et al. believed that the ultraviolet light emitted by plasma can induce the generation of massive photogenerated electrons and holes over the TiO_2_ coating surface, thereby promoting O_3_ synthesis based on the photocatalytic effect [64,116].

## 4. O_3_ Synthesis Performance of Coupled Systems

### 4.1. Packing Catalyst

Numerous studies have demonstrated the improvement of O_3_ synthesis through placing the packing catalyst into a DBD reactor [53,62,94,109,112]. Schmidt-Szałowski et al. [123] fabricated a cylindrical packed DBD O_3_ reactor by packing irregular SiO_2_ particles (1.25–3.2 mm in size) into the discharge gap. The results demonstrated that packing the SiO_2_ particle significantly accelerated the O_3_ synthesis reactions, leading to a marked increase in O_3_ concentration. Also, Schmidt-Szałowski et al. [95,124] investigated the effects of porous SiO_2_ and quartz glass particles, which share similar chemical compositions but exhibit distinct structural and porosity differences, on O_3_ synthesis in a cylindrical reactor. Their study revealed that particle size significantly influenced O_3_ synthesis performance: packing larger particles (0.5–0.8 mm) resulted in higher O_3_ concentration and production compared to packing smaller particles (0.16–0.315 mm). Furthermore, it was found that porous SiO_2_ outperformed quartz glass in elevating O_3_ synthesis, attributing this effect to the surface structure of material rather than its porosity. Murphy et al. [125] investigated O_3_ synthesis in a DBD packed with glass spheres and observed that the presence of these beads reduced the breakdown voltage. Furthermore, by comparing O_3_ production under different feed gases (air vs. O_2_), they found that the O_3_ concentration generated in pure O_2_ was three to four times higher than that in air. Jodzis et al. [106] researched O_3_ synthesis using O_2_-N_2_ mixtures with O_2_ content ranging from 20% to 100%. They found that the reactor packed with SiO_2_ consistently achieved higher O_3_ concentrations than the unpacked reactor. This enhancement was particularly pronounced when air (20%O_2_-N_2_) served as the feed gas. Under the same conditions, the SiO_2_-packed reactor produced O_3_ concentrations that were 20–40% higher than those from the unpacked reactor. The experimental results of Chen et al. [111] showed that packing Al_2_O_3_ particle provided a superior enhancement effect on O_3_ synthesis contrast to packing glass particles. Specifically, when packing 2 mm Al_2_O_3_ particles, the reactor attained peak-performance metrics: an O_3_ concentration of 61 g/m^3^, a production of 3.7 g/h and an efficiency of 173 g/kWh. These values corresponded to an 8-fold increase in concentration and a 12-fold increase in efficiency relative to a conventional unpacked reactor. The study also revealed that for both oxide materials, optimal particle size existed for packing (neither excessively large nor small particles were most beneficial for enhancing O_3_ synthesis), indicating a non-linear relationship between the packed particle size and O_3_ synthesis performance.

In addition, Huang et al. [126] combined hybrid discharge with γ-Al_2_O_3_ spheres to boost O_3_ synthesis. The experimental results showed that after packing γ-Al_2_O_3_ spheres, the highest O_3_ efficiency of the coupled system is 127.1 g/kWh, which is approximately twice that of the unpacked reactor. Pekárek et al. [127] found that packing TiO_2_ particles significantly promoted O_3_ synthesis. The highest O_3_ concentration and efficiency achieved with packing were 1100 ppm and 80 g/kWh, respectively, which were 1.4 times and 1.6 times higher than those without packing. Dwivedi et al. [128] explored the effects of packing a 13 X spherical molecular sieve, Pyrex particles, Pyrex cotton and porous TiO_2_ on O_3_ concentration and efficiency of DBD in argon–oxygen mixed gas. It was found that packing Pyrex beads, Pyrex wool and porous TiO_2_ was more conducive to O_3_ synthesis than packing a 13 X spherical molecular sieve. This is mainly attributed to the larger specific surface area of Pyrex beads and Pyrex wool (which improves the surface O_3_ synthesis reaction) and the larger dielectric constant of porous TiO_2_ particles (which increases the electric field strength between particles and then favors O_3_ synthesis reaction). Zeng et al. [62] conducted O_3_ synthesis by packing quartz fibers and SiO_2_-loaded quartz fibers, respectively. Compared with no packing, the O_3_ concentration and efficiency of packing pure fibers increased by 10.31% and 14.22%, and the packing of SiO_2_-loaded fibers increased to 22.26% and 35.49%, respectively.

However, for packed reactors, two drawbacks make them unfriendly for O_3_ applications. On the one hand, the dense packing of catalysts can easily increase gas resistance, which is not conducive to timely heat dissipation and can increase the thermal decomposition of O_3_. So, O_3_ workers usually add cooling devices (recirculating cooling water, heat dissipation components, etc.) to packed-bed reactors to reduce the thermal decomposition of O_3_. On the other hand, due to the need to pack catalysts, reactors with larger discharge gaps are more suitable. O_3_ workers believe that replacing catalyst packing with a catalyst coating/film can effectively avoid the above drawbacks.

### 4.2. Nanocatalyst Coating/Film

**Figure 7:**
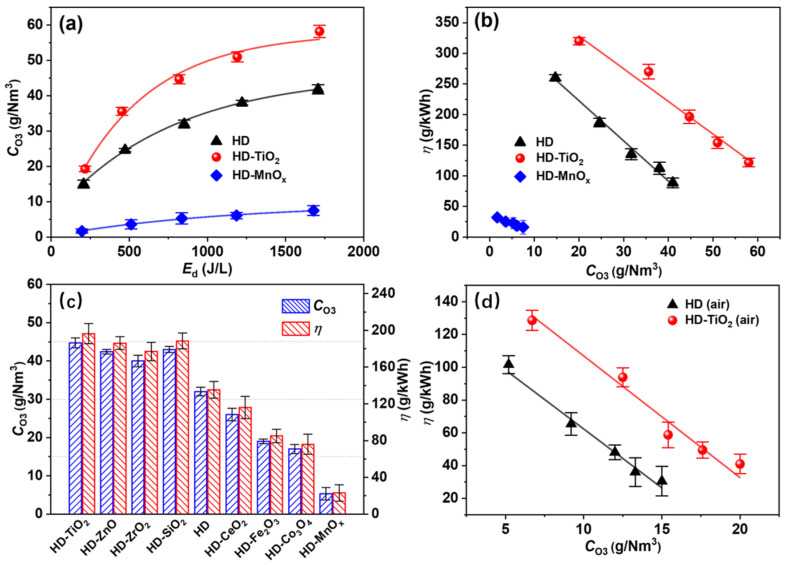
O_3_ concentration as a function of energy density (**a**) and O_3_ energy yield as a function of O_3_ concentration (**b**) for the reactor coupled with TiO_2_ and MnO*_x_* coating, respectively, in pure O_2_ discharge; effect of various oxide coatings on O_3_ synthesis in pure O_2_ discharge (**c**); effect of TiO_2_ coating on O_3_ synthesis in air discharge (**d**) (Reproduced with permission from reference [67]).

**Figure 8:**
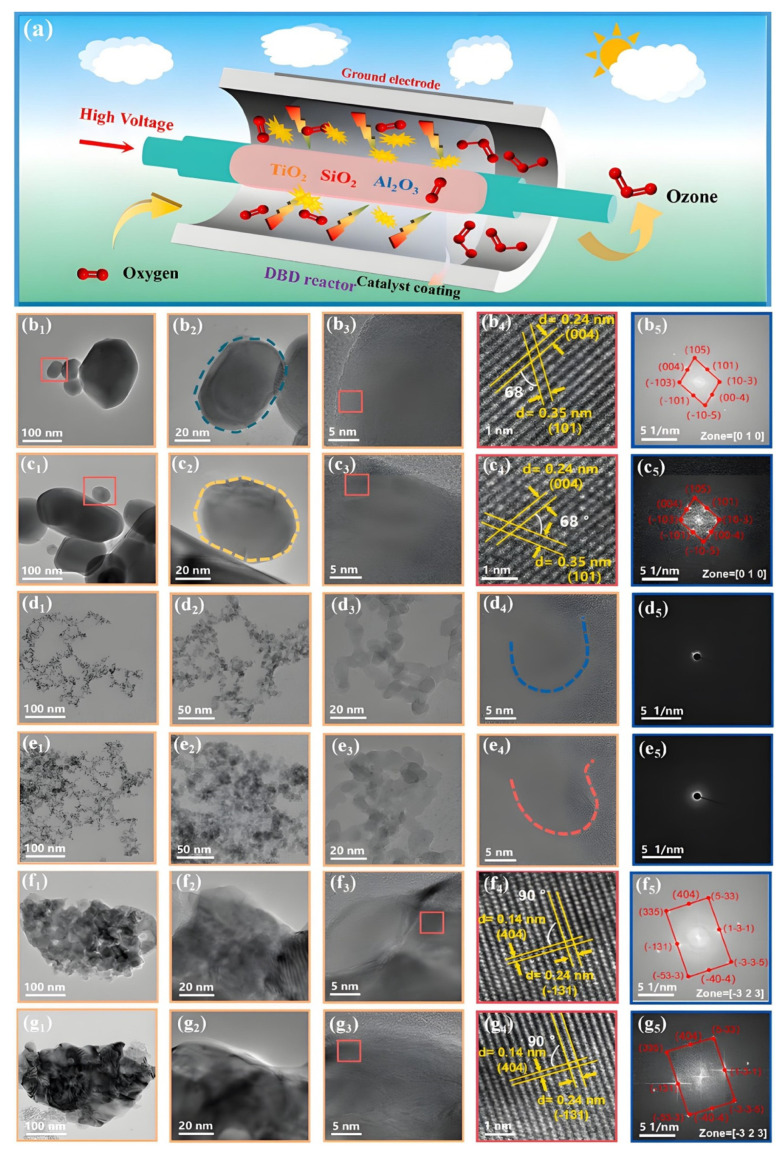
Mechanism diagram of the coupled system (**a**); SEM images of TiO_2_ film before discharge (**b_1_**–**b_5_**), TiO_2_ film after discharge (**c_1_**–**c_5_**), SiO_2_ film before discharge (**d_1_**–**d_5_**), SiO_2_ film after discharge (**e_1_**–**e_5_**), Al_2_O_3_ film before discharge (**f_1_**–**f_5_**) and Al_2_O_3_ film after discharge (**g_1_**–**g_5_**). The substance inside the red box is TiO_2_ nanoparticle, and the blue and red dashed boxes are respectively SiO_2_ nanoparticles before and after discharge (Reproduced with permission from reference [61]).

**Figure 9:**
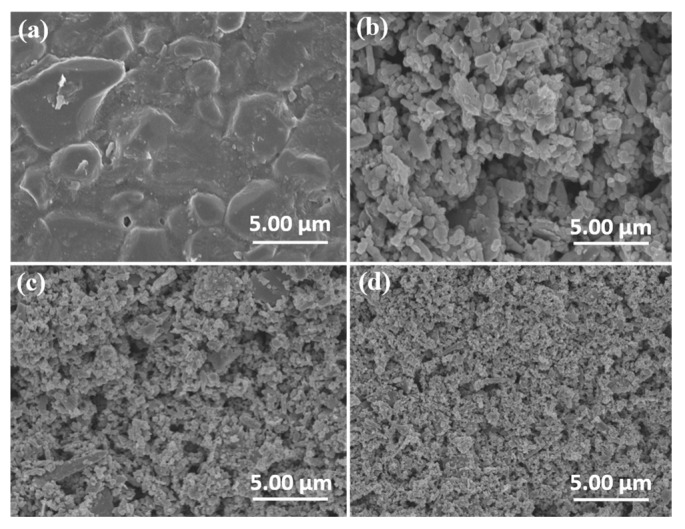
SEM images of Al_2_O_3_ dielectric plate (**a**) and ZnO coatings made of 50 nm (**b**) and 20 nm (**c**,**d**) nano-powders, respectively, where (**d**) is the image after discharge and the others are before discharge (Reproduced with permission from reference [58]).

Compared with catalyst packing, the technology of using nanocatalyst coatings/film to enhance DBD O_3_ synthesis has developed later but demonstrates tremendous application potential. Wei et al. [129] coupled a SiO_2_ film made by the sol-gel method into DBD and found that, the thicker the film, the more unfavorable it is for O_3_ synthesis, because thick film is not conducive to heat dissipation. The presence of SiO_2_ film increased the O_3_ synthesis concentration and efficiency of DBD by 7.1% and 72.6%, respectively, under pure O_2_ discharge. Coupling different nanocatalyst coatings with a hybrid discharge reactor, the effect of nanocatalyst material on discharge characteristics and the performance of O_3_ synthesis are systematically studied in pure O_2_ and air by Li et al. (see Figure 7) [67]. The comprehensive results of O_3_ synthesis, catalyst characterization and discharge characteristics diagnosis indicated that O_3_ synthesis strongly depends on the features of nanocatalysts. The single-valent nanocatalysts (TiO_2_, SiO_2_, ZnO, ZrO_2_, etc.) can enhance surface reactions, thus promoting O_3_ production because of their large surface area but low concentration of oxygen vacancies. This positive impact of the materials for these catalysts on O_3_ synthesis was also found by Shi et al. using a cylindrical DBD–catalyst coupled system (see Figure 8a) [61,96]. Meanwhile, their results also demonstrated that the discharge plasma does not destroy the crystal lattice structure of these nanocatalysts, but it can make the surface of nanocatalyst film rough (see Figure 8b_1_–b_5_,g_1_–g_5_), suggesting that the plasma process is beneficial for increasing the catalyst surface area and the gas–solid interface O_3_ synthesis reaction. In contrast, the multivalent nanocatalysts (MnO*_x_*, Co_3_O_4_, Fe_2_O_3_, CeO_2_, etc.) possess large numbers of oxygen vacancies and redox couples, which mainly lead to the fast O_3_ decomposition via surface catalytic reactions over oxygen vacancies and redox couples, thus resulting in a decline in O_3_ synthesis. Hence, selecting appropriate nanocatalyst coatings can effectively regulate the discharge O_3_ synthesis. Among all nanocatalyst coatings (ZnO, ZrO_2_, SiO_2_, MnO*_x_*, Co_3_O_4_, etc.) used by Li et al, coupling TiO_2_ coating enabled the discharge reactor to achieve the highest O_3_ concentration (19.3–58.2 g/Nm^3^) and efficiency (320.0–121.8 g/kWh), improving by approximately 41% and 38%, respectively, in comparison with the discharge alone [67]. Furthermore, Li et al. further demonstrated that reducing the particle size of the nanocatalyst (ZnO) for preparing nanocatalytic coatings can enable the coating to achieve a high specific surface area (see Figure 9), thereby facilitating the discharge O_3_ synthesis of the coupled system [58]. Due to the increased plasma density when reducing the discharge gap (high plasma density facilitates the interaction and physicochemical reactions between coating/film and active species), a superior O_3_ synthesis performance was obtained at a relatively narrow discharge gap for the coupled system of DBD with a ZnO coating [38,58].

The photocatalytic effect of the oxide catalyst coating/film is recognized as a main factor to enhanced O_3_ synthesis by some researchers. Mikeš et al. [130] investigated the effect of a photocatalyst coating of TiO_2_, ZnO, BaTiO_3_ and WO_3_ on O_3_ synthesis in air discharge. The results suggested that a TiO_2_, ZnO and BaTiO_3_ coating can all assist O_3_ synthesis of DBD, while WO_3_ has no effect on O_3_ synthesis. Compared to DBD without photocatalyst coating, the O_3_ concentration using a TiO_2_, ZnO and BaTiO_3_ coating increased by about 30%, 19% and 19%, respectively. Capp et al. [64] found that, in a BaTiO_3_-packed DBD reactor, when depositing TiO_2_ coatings onto the surface of BaTiO_3_ particles via magnetron sputtering, the presence of TiO_2_ affects the plasma chemistry through acting as an atomic oxygen sink, photocatalytic formation of O_2_^−^, and modification of the dielectric constant of the BaTiO_3_ particulates, then improving the O_3_ synthesis. Pekárek et al. [65] studied the effect of a TiO_2_ layer covering various regions of the fence-like active electrode on the O_3_ concentration (covering only the strips, the region between the strips, and all active electrode, respectively). Covering only the strips of the fence-like active electrode with the TiO_2_ layer obtains the highest O_3_ synthesis compared to other investigated cases. Furthermore, Pekárek et al. [66] employed a drop-coating method to apply a photocatalyst film of TiO_2_ or ZnO onto the inner surface of the dielectric plate opposite the strip electrode in a conventional surface (S-) DBD reactor; the structure is shown in Figure 4b,c. The results demonstrated that TiO_2_ and ZnO coatings exhibited nearly identical enhancement effects on O_3_ synthesis in the SDBD reactor under air-discharge conditions. At an O_3_ concentration of 2 g/Nm^3^, the corresponding O_3_ efficiency reached approximately 113 g/kWh, representing an improvement of 11% and 15% compared to the SDBD without catalyst film, respectively. Moreover, Lu et al. [116] employed the sol-gel method to deposit a TiO_2_ film onto the surface of an Al_2_O_3_ dielectric layer, achieving a synergistic enhancement effect of photocatalyst TiO_2_ and a volume (V-) DBD on O_3_ synthesis in air discharge. Under an AC voltage of 13 kV, the O_3_ concentration and O_3_ efficiency of the coupled system increased by 56% and 38%, respectively, compared to the VDBD alone. However, in air discharge, since the plasma process simultaneously emits ultraviolet and visible light, it is difficult to distinguish whether photocatalytic effects or surface reactions play a dominant role in enhancing O_3_ synthesis when the photocatalysts (TiO_2_, ZnO, etc.) are used. Although most scholars attribute it to the photocatalytic effect, we believe it should be the result of the combined action of the photocatalytic effect and surface reactions. So far, there has been no research reported on this aspect yet. To address this question, the following experiments could be conducted in the future. Using oxides with surface structures similar to TiO_2_ but lacking photocatalytic activity—such as SnO_2_ (rutile structure, band gap of ~3.6 eV) and ZrO_2_ (rutile-related structure, band gap of ~5.0 eV)—to replace TiO_2_ in air discharge O_3_ synthesis can eliminate the interference of photocatalytic effects. By comparing the results with those obtained using TiO_2_, maybe the respective contributions of photocatalytic effects and surface reactions to O_3_ synthesis can be discerned.

From Table 2, it can be seen that the existence (packing and coating/film) of catalysts is beneficial for the improvement of DBD O_3_ synthesis, and the single-valent oxides of TiO_2_, SiO_2_, Al_2_O_3_ and ZnO are the most used catalysts. Due to different experimental conditions, it is difficult to make a direct and accurate comparison of the O_3_ generation performance between the coupling methods (packing and coating/film) of the catalyst and DBD. However, based on a comprehensive analysis of the O_3_ synthesis results, the packing method appears to perform better in air discharge, whereas the coating/film method shows superior performance in pure O_2_ discharge. The method of adding nanocatalyst coating/film into DBD can effectively avoid the disadvantages of the method of packing catalysts mentioned in Section 4.1 (high heating effect and wide discharge gap) during the O_3_ synthesis process [58,67]. Because the coating/film adhered to the surface of dielectric plate/layer is commonly very thin (~nm or ~µm) and the space volume it occupies can be ignored, the presence of the coating/film hardly affects the volume fraction of the discharge gap and gas-flow rate in DBD [60]. In view of this, even under an extremely narrow discharge gap, an effective combination and interaction of catalyst and discharge, as well as the catalytic enhancement effect of catalyst on O_3_ synthesis during the discharge process, can still be achieved via using the nanocatalyst coating/film [58,67].

## 5. Advanced Plasma Parameter Detection Techniques

In a DBD–catalyst coupled system, the strong interaction between plasma and catalyst influences their respective characteristics [60,62]. Catalysts can modify plasma physicochemical reactions by altering discharge/plasma characteristics, including gap electric-field strength, fundamental plasma parameters, and reactive oxygen species density, which in turn induce changes in gas–solid interface reactions occurring on the catalyst surface [57,58]. Therefore, whether in gas-phase reactions or gas–solid interface reactions, the type and concentration of high-energy active species in the plasma serve as critical parameters governing O_3_ synthesis. Although extensive research has been conducted on the synergistic enhancement of O_3_ synthesis by plasma and catalyst and reached a consensus on the underlying mechanism [61,67,112], systematic measurements of plasma parameters (such as electron temperature and density, as well as oxygen atom density) in O_3_ synthesis with the presence of catalyst remain scarce in reported studies. Systematic determination of plasma parameters facilitates further in-depth research and analysis of the enhancement mechanism of coupling catalysts on O_3_ synthesis.

Plasma-parameter measurement involves various methods, including Langmuir probe [132], microwave interferometry [133], laser Thomson scattering [134] and optical emission spectroscopy [135]. The Langmuir probe method typically requires inserting a metal probe into the plasma and applying a scanning voltage to measure the current–voltage curve, thereby determining the electron temperature in the plasma. The method uses simple and inexpensive equipment, but its intrusive measurement nature not only disturbs the plasma, but also exposes the metal probe to contamination or damage from reactive gases (especially corrosive gases), and may even induce sputtering on the probe surface, thereby affecting the accuracy of plasma-parameter measurement. Although microwave interferometry and laser Thomson scattering are both completely non-intrusive plasma-parameter measurement techniques, they are typically used for the determination of parameters in high-temperature and high-density plasmas, and require complex and expensive equipment. Microwave interferometry is suitable for diagnosing high-temperature plasmas confined by strong magnetic fields, while laser Thomson scattering is more commonly used for diagnosing large fusion plasmas.

Compared to other methods, optical emission spectroscopy (OES) is the most widely used non-intrusive diagnostic technique for low-temperature plasmas. The technique is not only mature but also possesses excellent spatial and temporal resolution, enabling the acquisition of two-dimensional or even three-dimensional distribution maps of plasma parameters through point-by-point scanning or imaging spectroscopy, which is crucial for studying plasma inhomogeneity and discharge channels. In addition, OES allows for the simultaneous determination of multiple key plasma parameters through the analysis of one or several spectral lines. Therefore, OES is highly suitable for the measurement of parameters in low-temperature plasmas during the O3 synthesis process.

### 5.1. Optical Emission Spectroscopy

OES is a powerful technique for plasma-parameters measurement, such as rotational temperature (*T*_rot_), vibrational temperature (*T*_vib_), electron-excitation temperature (*T*_exc_), electron density (*n*_e_) and atomic oxygen density ([*O*]) [39,135]. These plasma parameters are often used to characterize the characteristics of the discharge plasma. Generally, *T*_vib_ and *T*_exc_ can reflect the non-thermal plasma characteristics of DBD, which is the basis of O_3_ synthesis [39]. Since energetic electrons and O are the key factors involved in O_3_ synthesis reactions according to the O_3_ reaction mechanism, the physicochemical activity of the plasma can be judged based on the measured *T*_exc_, *n*_e_, and [*O*], thereby benefitting the kinetic analysis of ozone synthesis reaction.

**Figure 10:**
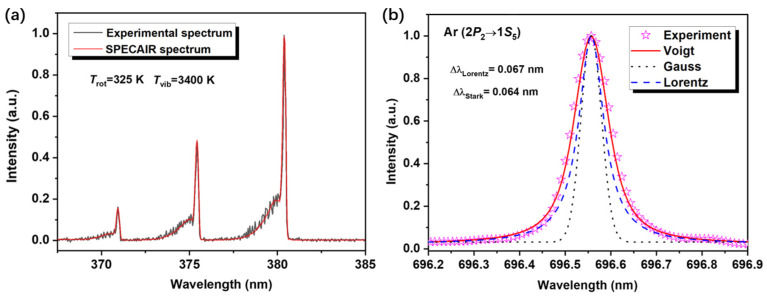
Typical measured N_2_ *C*^3^Π*_u_*→*B*^3^Π*_g_* spectrum and SPECAIR fit (**a**) and deconvolution plot of the Ar spectral line at a wavelength of 696.54 nm (**b**) (Reproduced with permission from reference [39]).

Plasma parameters are typically determined using the internal standard method based on emission spectroscopy; the calculation equations are summarized in Table 3. The spectra of N_2_ second positive (*C*^3^Π*_u_*→*B*^3^Π*_g_*) can be taken in a wavelength range of 365–385 nm via introducing 1–5% N_2_ into the discharge gas. Then, the simulations for N_2_ *C*→*B* can be conducted using the SPECAIR software (see Figure 10a) to estimate *T*_rot_ and *T*_vib_ [136,137]. Adding 1–10% Ar into the discharge gas is to obtain Ar emission spectral lines. According to a local equilibrium model, *T*_exc_ calculation can be acquired through the intensity of two lines of Ar atoms (763. 51 nm (2*P*_6_→1*S*_5_) and 772. 42 nm (2*P*_2_→1*S*_3_)) at the same ionization level (Equation R17) [39,138,139]. Meanwhile, the Ar spectral line at 696.54 nm (2*P*_2_→1*S*_5_), as a strong and isolated spectral line, can be adopted here to measure *n*_e_ based on the Stark broadening (∆*λ*_S_) characteristics of Ar atoms (see Figure 10b; Equations R18 and R19) [140,141,142]. Considering that the measurement of electron temperature (*T*_e_) in spectral analysis is difficult, *T*_exc_ is usually adopted instead of *T*_e_ to estimate *n*_e_ in practical measurements, because *T*_exc_ and *T*_e_ exhibit the same vibration trend during the discharge process. It should be noted that, due to the electron energy distribution deviating to some extent from the Maxwellian energy distribution, *T*_exc_ is slightly lower than *T*_e_ (the difference is less than 0.5 eV; they are equal under local thermodynamic equilibrium conditions). However, in discharge systems, the variation in *T*_exc_ can reflect the variation law of *T*_e_. Based on this, *T*_exc_ can be used in specific experiments to estimate *n*_e_ using Equations R18 and R19. Furthermore, [*O*] in the plasma can be detected based on the intensity ratio of O (844.6 nm, ^3^*P*→^3^*S*) and Ar (750.4 nm, 2*P*_1_→1*S*_2_) emission lines (Equation R20) [135,143,144].

### 5.2. Two-Photon Absorption Laser-Induced Fluorescence

**Figure 11:**
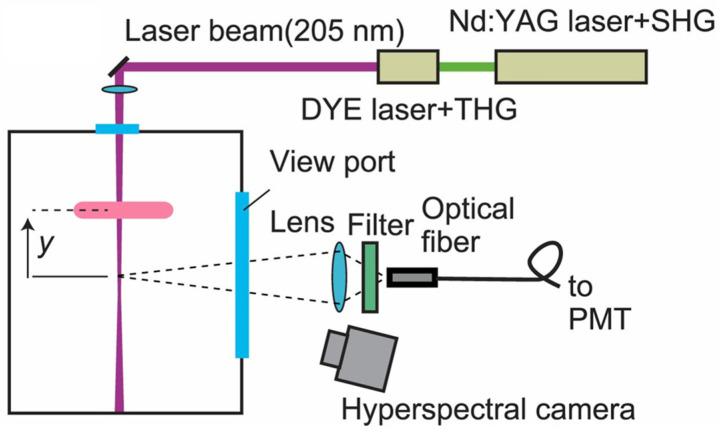
Experimental setup for TALIT (Reproduced with permission from reference [145]).

In addition, two-photon absorption laser-induced fluorescence (TALIF) serves as an effective diagnostic method for detecting [*O*] (as illustrated in Figure 11) [145,146]. Although the TALIF system is extremely complex and expensive (requiring a tunable dye laser or OPO laser), and suffers from drawbacks such as complicated calibration procedures (quenching correction, excitation cross-section, etc.) and high demands on laser power stability, it enables the measurement of absolute [*O*] in plasmas during O_3_ synthesis process, with high sensitivity allowing detection of very low absolute [*O*] [145]. Therefore, if laboratory conditions permit, combining OES and TALIF for [*O*] measurement can enhance the accuracy and scientific rigor of the experiment. This technique was first employed by Döbele et al. [146] in 2005 for the diagnosis of O in atmospheric-pressure plasma jets. They calibrated TALIF using Xe gas as a reference and obtained a preliminary spatial density distribution of O within the plasma jet, where the [*O*] near the nozzle reached as high as 2.8 × 10^15^ cm^−3^. Subsequently, numerous researchers adopted TALIF to measure [*O*] in plasma [147,148,149,150]. Ono et al. [151] studied the effect of different applied voltages and humidity on the decay rate of O in DBD via using TALIF technique. Knake et al. [152,153] investigated the influence of discharge power, oxygen content, and other factors on [*O*] within a plasma jet. Their study revealed that oxygen content significantly affected [*O*], with the highest [*O*] observed at an oxygen content of 0.5%. Burnette et al. [154] detected the absolute [*O*] between two copper electrodes with a working pressure of 100 Torr. Therefore, systematic detection of plasma parameters using the above methods can provide theoretical support for elucidating the mechanism of catalyst-enhanced O_3_ synthesis.

## 6. Conclusions and Prospects

This review mainly focuses on the research progress of the DBD–catalyst coupled system in the field of O_3_ synthesis, including the coupling method of DBD and catalysts, O_3_ synthesis performance of the coupled system, etc. Currently, with the increasing research interest in catalysts and their widespread application in various industrial fields, effectively coupling suitable catalysts with DBD is considered one of the most promising approaches for enhancing discharge O_3_ synthesis. Oxide catalysts are commonly used to construct DBD–catalyst coupled systems for improving O_3_ synthesis due to their low cost and relatively milder catalytic activity (less prone to catalyzing O_3_ decomposition during the plasma reaction process) compared to noble-metal catalysts. At present, the optimal ozone synthesis performance of the reactor with packed catalyst in air plasma (γ-Al_2_O_3_ sphere) is 0.96 g/Nm^3^ and 103 g/kWh, and in oxygen plasma (SiO_2_ particle) is 130 g/Nm^3^ and 91 g/kWh, respectively. For the reactor coupled with a catalyst coating, the performance reaches 19.3 g/Nm^3^ and 320 g/kWh in oxygen plasma (TiO_2_). However, it is worth noting that, according to the comprehensive analysis of existing research reports, the experimental studies from many scholars only reflect the enhancing effect of catalysts on DBD O_3_ synthesis. Despite some scholars mentioning the mechanism of catalyst-enhanced O_3_ synthesis, their descriptions remain rather general and insufficiently systematic. Simultaneously, no relatively direct evidence has been provided for the stated enhancement mechanisms, such as changes in key plasma parameters directly involved in O_3_ synthesis reactions (e.g., energetic electron density, oxygen atom density) before and after catalyst addition. In addition, although the use of catalysts effectively improves the efficiency of DBD O_3_ synthesis, it is still far below the theoretical value. Consequently, to promote the more mature development and application of O_3_ generators based on the DBD–catalyst coupled system, future research should still focus on the following two points:

(i) In order to gain insight into the enhancement mechanism, people can employ advanced plasma diagnostic techniques to measure the changes in critical plasma parameters within the DBD reactor upon catalyst addition, particularly electron temperature, electron density and oxygen atom density. Meanwhile, the discharge plasma morphology can be captured and observed using an ICCD high-speed camera and a high-resolution digital camera, enabling intuitive investigation and analysis of their micro- and macro-morphological changes before and after coupling different catalysts. Then, the various physical and chemical characterization techniques can be used to systematically characterize the coupled catalyst, including SEM, HR-TEM, BET, XRD, XPS, O_2_-TPD, EPR, UV-vis DRS and transient photocurrent measurements. Accordingly, the physicochemical properties of different catalysts can be compared before and after discharge, including crystalline phase structure, specific surface area, surface elements and their chemical states, oxygen vacancy concentration, surface oxygen species density, surface electron transfer rate, etc. Afterwards, based on the results of plasma diagnostics, catalyst characterization and O_3_ synthesis, a close relationship between the physicochemical properties of catalysts, discharge characteristics, plasma parameters, oxygen atom density and O_3_ synthesis performance can be established. It can provide not only strong support for elucidating the microscopic reaction processes of O_3_ synthesis in the DBD–catalyst coupling system, conducting reaction kinetics studies of O_3_ synthesis in the presence of catalysts and revealing the reaction mechanisms of catalyst-enhanced O_3_ synthesis of DBD in detail, but also theoretical guidance for the development of stable and efficient O_3_ generators based on the DBD–catalyst coupled system.

(ii) Adding an extra magnetic field (such as a neodymium iron boron (Nd_2_Fe_14_B) permanent magnet) around the DBD–catalyst coupled system for achieving a synergistic enhancement effect of the magnetic field, catalyst and DBD on O_3_ synthesis, is also a viable approach to further promote discharge O_3_ synthesis. Two magnets (Nd2Fe14B) are arranged in parallel at a certain distance to establish a magnetic field environment. The DBD reactor is placed in the magnetic field environment, thereby achieving the superposition of electric and magnetic fields. The superposition mode of the electric and magnetic fields (such as vertical or parallel superposition) can be adjusted by changing the position of the DBD reactor in the magnetic field, and the magnetic field strength can be adjusted by varying the distance between two magnets. The introduction of a magnetic field will inevitably alter the plasma parameters such as the electron temperature and density, oxygen atom density and gas temperature, thereby affecting the gas-phase reactions in the plasma region, the gas–solid interface reactions on the catalyst surface and then ozone synthesis performance of the reactor. So, by studying parameters such as the direction and strength of the magnetic field, discharge voltage and discharge frequency on the microscopic physicochemical reaction mechanism of ozone synthesis in the discharge system, a close correlation between magnetic field characteristics, catalyst characteristics, discharge characteristics, plasma parameters and O_3_ synthesis performance can be established. Using optimal experimental parameters is expected to achieve the maximum synergistic enhancement of O_3_ synthesis in the reactor by the magnetic field and catalyst, then further improve the concentration and efficiency of discharge O_3_ synthesis.

In summary, the application of catalysts in enhancing discharge O_3_ synthesis is still full of challenges, such as catalyst deactivation or physical loss during the discharge process, although a series of encouraging research results have been achieved by DBD–catalyst coupled system. However, we firmly believe that, through the combination of theoretical and experimental research in the future, these challenges will be gradually overcome, enabling catalysts to play an even greater role in accelerating O_3_ synthesis and their industrial application.

## Figures and Tables

**Table 1:** Main reactions during the O_3_ synthesis process [30,58,67].

	Reaction
R1	$e+O_{2}\to e+2O$
R2	$O+O_{2}+M\to O_{3}+M$
R3	$e+O_{3}\to O_{2}+O+e$
R4	$O+O_{3}\to 2O_{2}$
R5	$O_{2}+O_{3}\to O+O_{2}+O_{2}$
R6	$N+O_{3}\to \mathrm{NO}+O_{2}$
R7	$\mathrm{NO}+O_{3}\to NO_{2}+O_{2}$
R8	$NO_{2}+O_{3}\to NO_{3}+O_{2}$
R9	$N+O_{2}\to \mathrm{NO}+O$
R10	$N+\mathrm{NO}\to N_{2}+O$
R11	$N_{2}+O_{2}\to N_{2}+2O$
R12	$O_{2}+\mathrm{coating}\to {O_{2}}_{\left(\mathrm{ads}\right)}+O\to {O_{3}}_{\left(\mathrm{ads}\right)}\to O_{3}$
R13	$O+\mathrm{coating}\to O_{\left(\mathrm{ads}\right)}+O_{2}\to {O_{3}}_{\left(\mathrm{ads}\right)}\to O_{3}$
R14	$O_{2}^{-}+\mathrm{coating}\to {O_{2}}_{\left(\mathrm{ads}\right)}+e$
R15	$O^{-}+\mathrm{coating}\to O_{\left(\mathrm{ads}\right)}+e$
R16	${O_{3}}^{*}+M_{\left(\mathrm{coating}\right)}\to O_{3}$

*Note*: O_(ads)_, O_2(ads)_ and O_3(ads)_ represent the adsorbed O, O_2_ and O_3_, respectively; M_(coating)_ represents the third-body collision partner played by the catalyst coating.

**Table 2:** Summary of O_3_ synthesis for different DBD–catalyst coupled systems.

Coupled Catalysts		O_3_ Concentration (g/Nm^3^)	O_3_ Efficiency (g/kWh)	Feed Gas	References
Material	Size				
SiO_2_ particle	1.25–3.2 mm *	130	91	O_2_	[95,131]
Al_2_O_3_ sphere	0.7 mm *	80	210	O_2_ + 4%N_2_	[109]
γ-Al_2_O_3_ sphere	3–5 mm *	0.96	103.1	Air	[126]
MS	/	0.78	82.4	Air	[126]
TiO_2_ particle	3 × 4 mm *	2.4	30	Air	[127]
Glass bead	2 mm *	/	209	O_2_	[94]
3A MS	2 mm *	15.6	/	Air	[94]
Al_2_O_3_ pellet	2–10 mm *	61	173	O_2_	[111]
13X MS pellet	1 mm *	0.8	/	Air	[128]
Pyrex beads	2–3 mm *	1.5	/	Air	[128]
Pyrex wool	/	1.2	/	Air	[128]
TiO_2_ beads	10–20 mm *	1.6	/	Air	[128]
Quartz fiber	/	57.6	111.8	O_2_	[62]
SiO_2_-loaded fiber	/	61	126.61	O_2_	[62]
TiO_2_ coating	20 µm ^@^	19.3–58.2	320.0–121.8	O_2_	[67]
TiO_2_ coating	0.1 mm ^@^	2.2	240	O_2_	[96]
SiO_2_ film	/	/	212.8	O_2_	[61]
Al_2_O_3_ film	/	/	217.2	O_2_	[61]
SiO_2_ film	0.9 µm ^@^	90	50	O_2_	[129]
ZnO coating	20 nm ^@^	21.4	302	O_2_	[58]
TiO_2_ film	/	13.8	/	Air	[114]
TiO_2_ film	/	2.5	57	Air	[130]
TiO_2_ film	/	3.9	55	Air	[65,66]
ZnO film	/	3.6	54	Air	[66]

*Note*: “MS” represents molecular sieve; “*” represents the particle size of the packed catalyst, while “^@^” represents the thickness of the catalyst coating/film.

**Table 3:** Main equations for measuring plasma parameters [39,144].

	Equation
R17	$T_{\mathrm{exc}}=\frac{E_{1}-E_{2}}{k}/(\ln\frac{A_{1}g_{1}\lambda_{2}}{A_{2}g_{2}\lambda_{1}}-\;\ln\frac{I_{1}}{I_{2}})$
R18	$\lambda_{\mathrm{Stark}\;}=2\;\times \;\left[1+1.75\;\times \;10^{-4}n_{e}^{1/4}\alpha \;\times \;(1-0.0675n_{e}^{1/6}T_{e}^{-1/2})\right]\;\times \;10^{-16}wn_{e}$
R19	$w=1.796\;\times \;10^{-3}{T_{e}}^{0.3685}$
R20	$\frac{\left[O\right]}{\left[\mathrm{Ar}\right]}=\frac{{\mathrm{hv}}_{750}A_{\mathrm{ij}}^{2p1}}{{\mathrm{hv}}_{844}A_{\mathrm{ij}}^{3p}}\frac{\sum A_{\mathrm{ij}}^{3P}}{\sum A_{\mathrm{ij}}^{2p1}}\frac{I_{844}}{I_{750}}\frac{K_{\mathrm{Ar}}^{2p1}}{K_{O}^{3P}}$

## Data Availability

No new data were created or analyzed in this study. Data sharing is not applicable to this article.
